# Intraoperative application of regional cerebral oxygen saturation monitoring for geriatric patients in China: a survey

**DOI:** 10.3389/fmed.2023.1165821

**Published:** 2023-09-07

**Authors:** Jie Zhang, Haiyan Shen, Huiping Wang, Feng Xiao, Lu Deng, Xiang Chen, Yongqiu Xie

**Affiliations:** ^1^Clinical Nursing Teaching and Research Section, The Second Xiangya Hospital of Central South University, Changsha, Hunan, China; ^2^Operation Room, The Second Xiangya Hospital of Central South University, Changsha, Hunan, China; ^3^Department of Anesthesiology, The Second Xiangya Hospital of Central South University, Changsha, Hunan, China; ^4^Department of Anesthesiology, Xiangya Hospital of Central South University, Changsha, Hunan, China

**Keywords:** regional cerebral oxygen saturation, geriatric anesthesia, postoperative cognitive dysfunction, cerebral oximetry, descriptive study

## Abstract

**Background:**

Regional cerebral oxygen saturation (rSO_2_) monitoring is a real-time and non-invasive technique for estimating the balance of regional cerebral oxygen supply and consumption. Despite the growing popularity of this monitoring technique, data regarding outcome benefits remain sparse and contradictory. This study was conducted to explore the popularity and understanding of cerebral oxygen saturation monitoring during anesthesia in geriatric patients.

**Methods:**

An online self-report questionnaire was distributed in March 2021 to various hospitals in China for dissemination to anesthesiologists. Questions surveyed cerebral oximetry equipment and utilization, demographics, and clinical practice of participants.

**Results:**

In total, 447 anesthesiologists responded. Of these, 301 (67.3%) respondents reported that their hospitals were equipped with cerebral oximetry, which 274 anesthesiologists use during anesthesia. A high percentage of anesthesiologists chose to monitor rSO_2_ during cardiac surgery (77.4%, *n* = 212) and neurosurgery (40.5%, *n* = 111). Most anesthesiologists agreed that a 30% reduction from the rSO_2_ baseline requires intervention to avoid cerebral ischemia, mainly via elevating arterial pressure and fraction of inspired oxygen (FiO_2_). Of those without cerebral oximetry, 138 of 146 (94.5%) anesthesiologists were willing to monitor rSO_2_. In addition, 291 respondents believed that cerebral oxygen monitoring would help prevent postoperative cognitive dysfunction.

**Conclusion:**

Our survey indicated that the prevalence of cerebral oximetry remains relatively low, while almost all anesthesiologists expressed their willingness to use rSO_2_ monitoring in geriatric anesthesia. Heterogeneity in clinical practice was identified, indicating relevant knowledge gaps that should encourage further clinical research to optimize treatment.

## Introduction

Regional cerebral oxygen saturation (rSO_2_) monitoring, based on near-infrared spectroscopy (NIRS), is a real-time and non-invasive technique for estimating the balance of regional cerebral oxygen supply and consumption (1). The brain consumes the highest percentage of oxygen by weight and is very vulnerable to hypoxia. The imbalance of oxygen supply and consumption during the perioperative period in the brain will inevitably lead to adverse outcomes, especially in geriatric patients. Previous studies found that patients with substantial cerebral oxygen desaturation during anesthesia might develop an increased incidence of adverse perioperative outcomes, including postoperative cognitive dysfunction (POCD), major organ failure, and mortality (2, 3). Therefore, the proper management of brain oxygenation is essential to providing effective anesthesia care for geriatric patients.

Once brain hypoxia occurs in the perioperative period, in addition to brain aging and systemic comorbidities, elderly patients will likely experience accelerated functional decline of the brain and other organs (4, 5). The demanding nature of oxygen balance for geriatric patients might increase the incidence of disability related to anesthesia in the absence of rSO_2_ monitoring, which may seriously affect the long-term quality of life after surgery and increase the burden of care on the patients' family and social network (6, 7). Given that China's population is aging, the proportion and number of geriatric patients will continue to increase rapidly. Therefore, rSO_2_ monitoring during anesthesia in geriatric patients may be an option that can be considered in clinical practice to reduce adverse outcomes (8).

Although there are hundreds of reports outlining numerous plausible uses for rSO_2_ monitoring during anesthesia, it remains difficult to implement, even at tertiary hospitals in China because of the growing number of geriatric surgeries and the shortage of devices. There are no reports on the use of rSO_2_ monitoring to manage anesthesia in geriatric patients in China. Hence, the present survey was conducted to explore the popularity of cerebral oxygen saturation monitoring and describe the current understanding of cerebral oximetry-guided geriatric anesthesia at hospitals in China.

## Materials and methods

This study was approved by the Medical Ethics Committee of the Second Xiangya Hospital Central South University (No. 2019S402). Informed consent was obtained from all participants. No incentives were provided for survey participation.

### Participants

The participants were recruited from various hospitals in China through convenience sampling. According to a recent report (9), there are more than 90,000 anesthesiologists in China. The sample size (n) was calculated using Raosoft software (available online at http://www.raosoft.com/samplesize.html). Keeping the confidence interval at 95% and the error margin at 5%, the representative sample size was calculated as 383 based on the population size of 90,000. Considering the non-response rate of 10%, the total minimal sample size should be set at 421. A total of 483 anesthesiologists, who demonstrated a full understanding of the purpose of the study and methods, were enrolled. The inclusion criteria for the participants were as follows: (1) being an anesthesiologist for more than 1 year in China; (2) being able to speak and read Chinese; and (3) voluntarily agreeing to participate in this study. We excluded individuals who had not worked in a clinical position for at least 3 years.

### Questionnaire development

Before the survey started, the literature related to cerebral oxygen saturation monitoring was searched in public databases such as PubMed, EMBASE, and Google Scholar. After the literature review, a preliminary questionnaire was sent to six anesthesiologists for editing and modification to ensure integrity and clarity. The final questionnaire was improved by clarifying the description of the clinical information and available answers and changing the order of the questionnaire. All of them were closed-ended questions (multiple-choice or checkbox).

**Figure 1:**
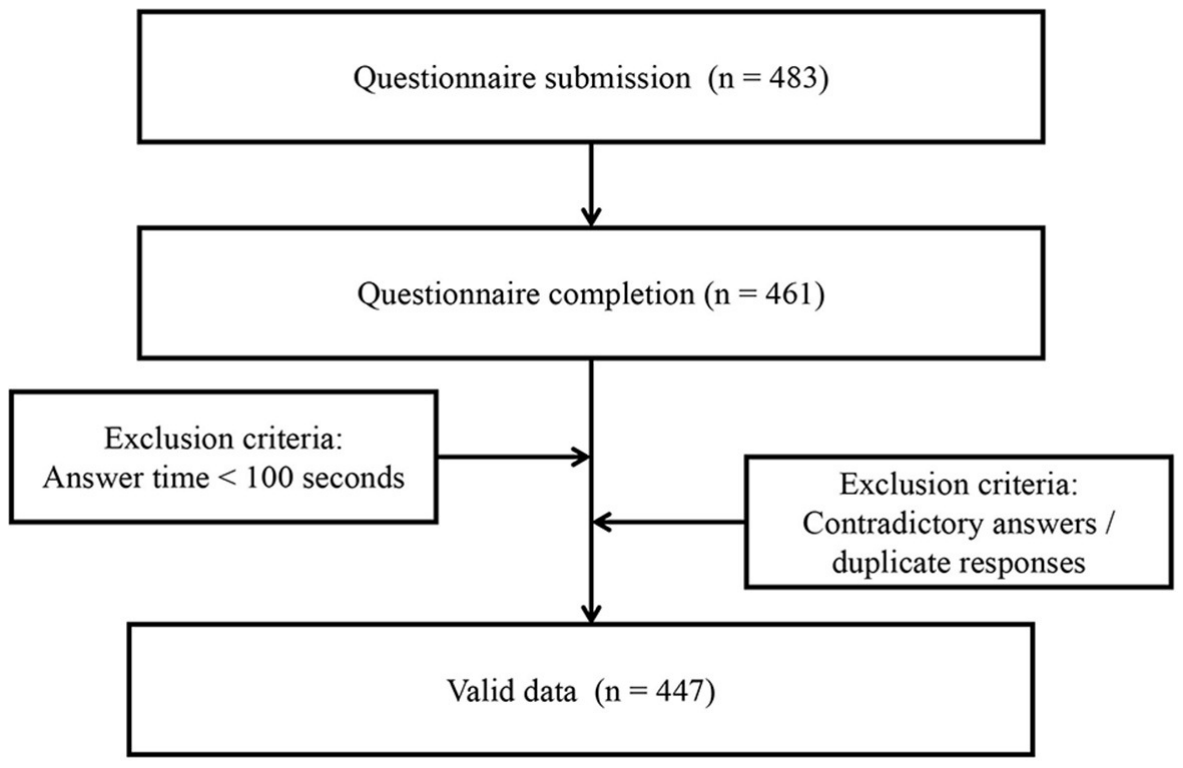
Flowchart for investigative procedures.

The electronic questionnaire was first distributed to the vice chairpersons of the Youth Group of the Anesthesia Branch of the Chinese Medical Association (CSA) and then sent to the anesthesiologists in their respective hospitals. The questionnaire was distributed to anesthesiologists via WeChat, and a single reminder letter was sent 1 week later if the anesthesiologists had not yet answered. After the participants signed the written informed consent forms, the complete questionnaire for the online survey was sent to anesthesiologists. Of the 483 questionnaires sent, 461 were completed. We carefully examined the questionnaires and excluded those with completion times of <100 s and those with contradictory answers or duplicate responses. Thus, 447 questionnaires from 23 administrative regions were included in the final analysis (Figure 1). A response rate of 92.5% is ideal for further data analysis (10). Participant information was coded electronically for data storage, and the investigator's computer was password-protected to prevent unauthorized access. Participants were not asked to provide their personal information, such as their name and the hospital where they worked.

### Data collection procedures

Data were collected through online self-report questionnaires via WeChat from 19 March to 25 April 2021. Details of basic characteristics, including sex, education level, annual number of anesthesia procedures for geriatric patients, hospital level, hospital type, years of employment, and professional title, were collected from a self-report questionnaire. The hospital level was divided into tertiary and primary hospitals (11). The hospital type was divided into general and specialized hospitals. Years of employment were divided into four groups: within 5 years, 6–10 years, 11–20 years, and over 20 years. The professional title was divided into five categories, including junior (medical assistant and resident), intermediate (attending physician), deputy senior (associate chief physician), and senior (chief physician), which are different from rankings used in Europe and America (9).

Data on rSO_2_ monitoring were collected based on the following questions: “Is your hospital's surgical anesthesia department equipped with cerebral oximetry?”, “Do you monitor cerebral oxygen saturation during anesthesia for geriatric patients (age ≥ 60 years old)?”, “If your hospital equipped with cerebral oximetry, would you consider using it during anesthesia?”, [sic] and “What cutoff value of the cerebral oxygen saturation declined from baseline during anesthesia should be intervened to avoid cerebral ischemia?”.

### Statistical analysis

Data analysis was performed using the SPSS Statistics software version 21.0 (IBM; Armonk, New York, USA). Descriptive statistics were used to summarize the sample characteristics and study variables. Categorical variables were summarized with frequencies and percentages.

## Results

### Demographic characteristics of respondents

The demographic characteristics of the respondents are shown in Table 1. Most anesthesiologists came from tertiary hospitals (87.9%, *n* = 393) and general hospitals (93.7%, *n* = 419). Generally, a total of 217 (48.5%) anesthesiologists had a master's degree, and 183 (40.9%) were physicians-in-charge. All respondents were evenly distributed across the time period of their working experience as anesthesiologists. Moreover, a total of 301 (67.3%) anesthesiologists reported that their hospitals were equipped with cerebral oximetry.

**Table 1:** Sociodemographic characteristics of the study sample.

**Sociodemographic**	**Frequency (%)**
Total sample	447 (100%)
**Sex**	
Male	210 (47.0%)
Female	237 (53.0%)
**Education level**	
Bachelor degree	133 (29.8%)
Master degree	217 (48.5%)
Doctoral degree	97 (21.7%)
**Level of hospital** ^a^	
Tertiary	393 (87.9%)
Secondary	54 (12.1%)
**Type of hospital**	
General hospital	419 (93.7%)
Specialized hospital	28 (6.3%)
**Years of working as anesthesiologists**	
≤ 5 years	106 (23.7%)
6-10 years	117 (26.2%)
11-20 years	111 (24.8%)
> 20 years	113 (25.3%)
**Professional title**	
Junior	70 (15.7%)
Intermediate	183 (40.9%)
Deputy senior	118 (26.4%)
Senior	61 (13.6%)
Others	15 (3.4%)
**Own cerebral oximetry**	
Yes	301 (67.3%)
No	146 (32.7%)

^a^a tertiary hospital is a comprehensive, referral, general hospitals at the city, provincial or national level with a bed capacity exceeding 500. A secondary hospital is one that tend to be affiliated with a medium size city, county or district and contain >100 beds, but <500.

### The willingness to monitor cerebral oxygen saturation

For respondents who reported owning cerebral oximetry, 274 of 301 (91.0%) anesthesiologists claimed that they sometimes choose to monitor cerebral oxygen saturation during geriatric anesthesia depending on the physiology of patients and type of surgery, while 27 (9%) anesthesiologists never monitor cerebral oxygen saturation (Table 2). For the anesthesiologists who did not own the cerebral oximetry, 138 of 146 (94.5%) participants were willing to monitor rSO_2_ during anesthesia, and the proportion increased with years of working as anesthesiologists.

**Table 2:** Sociodemographic for participants who are willing to monitor cerebral oxygen saturation with the present of cerebral oximetry or not.

**Variables**	**Own devices (*n* = 274)**	**Do not own devices (*n* = 138)**
**Sex**		
Male	115 (42.0%)	80 (58.0%)
Female	159 (58.0%)	58 (42.0%)
**Education level**		
Bachelor degree	34 (12.4%)	89 (64.5%)
Master degree	157 (57.3%)	42 (30.4%)
Doctoral degree	83 (30.3%)	7 (5.1%)
**Hospital levels**		
Tertiary hospital	269 (98.2%)	93 (67.4%)
Primary hospital	5 (1.8%)	45 (32.6%)
**Type of hospital**		
General hospital	268 (97.8%)	117 (84.8%)
Specialized hospital	6 (2.2%)	21 (15.2%)
**Years of working as anesthesiologists**		
≤ 5 years	77 (28.1%)	21 (15.2%)
6–10 years	79 (28.8%)	29 (21.0%)
11–20 years	62 (22.6%)	40 (29.0%)
>20 years	56 (20.4%)	48 (34.8%)
**Professional title**		
Junior	47 (17.2%)	18 (13.0%)
Intermediate	116 (42.3%)	52 (37.7%)
Deputy senior	63 (23.0%)	42 (30.4%)
Senior	37 (13.5%)	23 (16.7%)
Others	11 (4.0%)	3 (2.2%)
**Number of geriatric anesthesia per year**		
≤ 200	124 (45.3%)	84 (60.9%)
>200	150 (54.7%)	54 (39.1%)

### Clinical practice of cerebral oximetry

**Figure 2:**
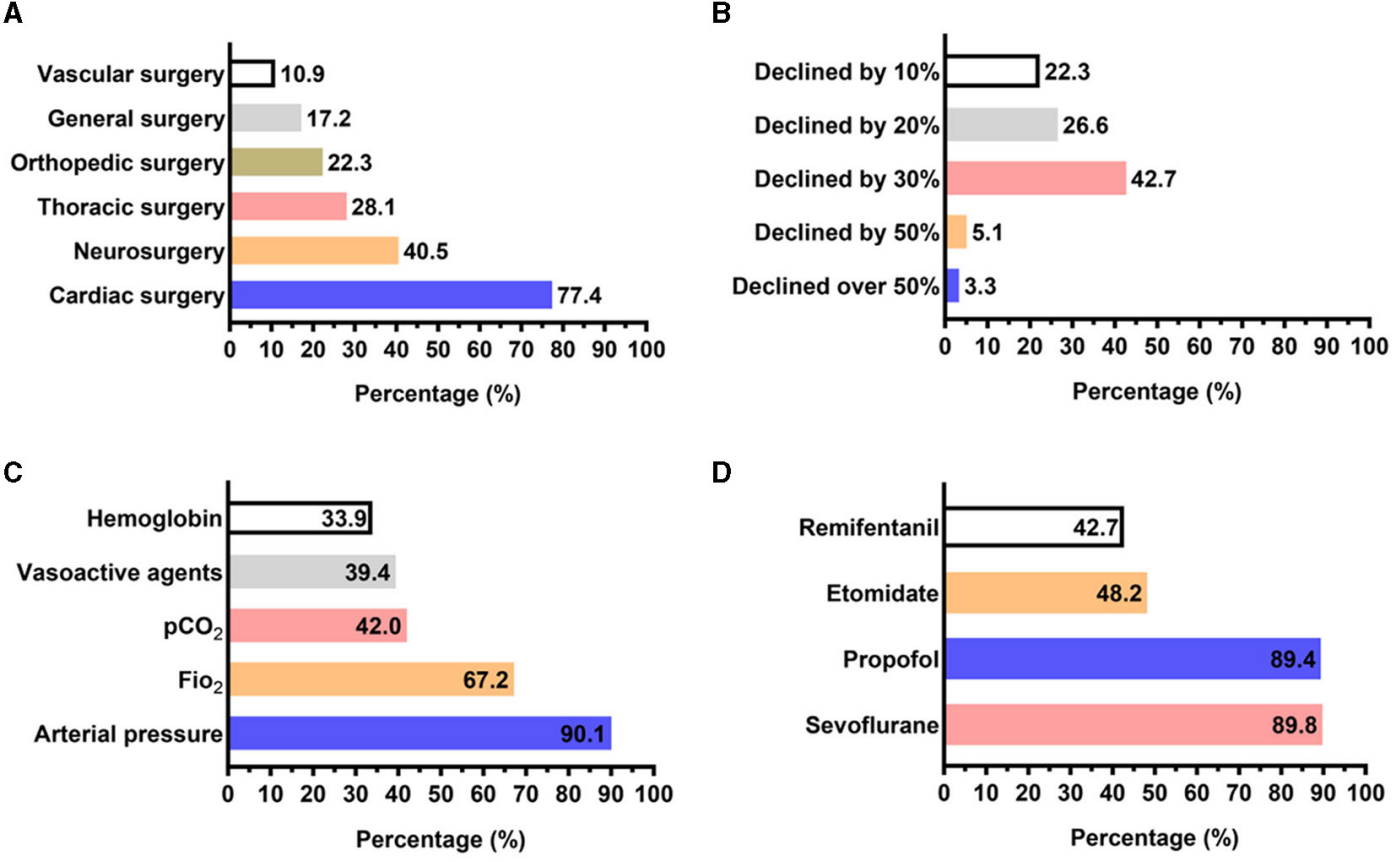
Answer distribution on the clinical practice and cutoff value of cerebral oxygen saturation monitoring in respondents who owned cerebral oximetry. **(A)** Answer distribution on which surgery the respondents would monitor cerebral oxygen saturation. **(B)** Answer distribution of the question: “what cutoff value of the cerebral oxygen saturation declined from baseline during anesthesia should be intervened to avoid cerebral ischemia?”. **(C)** Answer distribution of the question: “which intervention would be chosen to improve hypoxia if cerebral oxygen saturation declined?”. **(D)** Answer distribution of the question: “Which anesthetic could be guided during cerebral oxygen saturation monitoring?”.

For anesthesiologists equipped with devices, a high percentage of anesthesiologists chose to monitor rSO_2_ during cardiac surgery (77.4%, *n* = 212) or neurosurgery (40.5%, *n* = 111), rather than thoracic surgery (28.1%, *n* = 77), orthopedic surgery (22.3%, *n* = 61), and general surgery (17.2%, *n* = 47) (Figure 2A). Most of the anesthesiologists (42.7%, *n* = 117) stated they would take appropriate measures for increasing cerebral oxygen saturation to avoid cerebral ischemia when rSO_2_ declines by 30% from the baseline value (Figure 2B). A total of 247 (90.1%) anesthesiologists recognized that the most direct way to increase brain oxygen saturation is to elevate mean arterial pressure, followed by elevating FiO_2_ (67.2%, *n* = 184), decreasing partial pressure of carbon dioxide (PaCO_2_) (if <35 mmHg) (42.0%, *n* = 115), and providing vasoactive agents (39.4%, *n* = 108) and hemoglobin (33.9%, *n* = 93) (Figure 2C). In addition, most of the anesthesiologists believe that sevoflurane (89.8%, *n* = 246) and propofol (89.4%, *n* = 245) can be guided by a cerebral oxygen saturation monitor (Figure 2D).

### Association between rSO_2_ and risk of POCD

**Figure 3:**
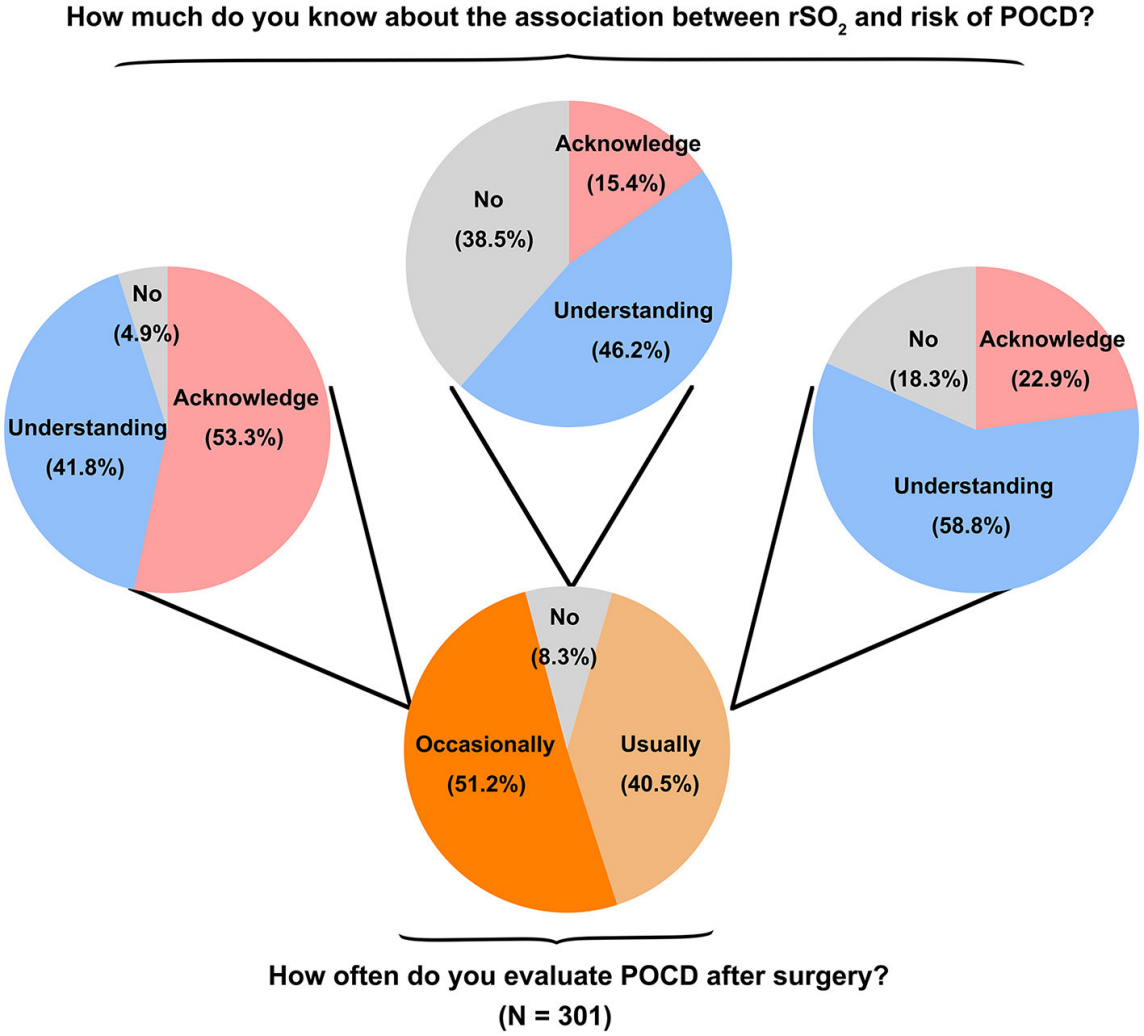
Summary of POCD evaluation and understanding the association between cerebral oxygen saturation monitoring and risk of POCD in respondents who owned cerebral oximetry.

Questions related to the POCD indicated that 122 of 301 (40.5%) respondents from the hospitals equipped with cerebral oximetry usually evaluated the POCD for geriatric patients after surgery, and 153 (50.8%) anesthesiologists did it sometimes (Figure 3). Notably, 65 of 122 (53.3%) anesthesiologists who usually evaluated POCD after surgery well understood the association between cerebral oxygen saturation and risk of POCD, but only 35 of 153 (22.9%) anesthesiologists who occasionally evaluated POCD after surgery well understood it. However, most respondents (96.7%, *n* = 291) considered that cerebral oxygen saturation monitoring could help reduce the incidence of POCD.

## Discussion

The present study was designed to investigate the popularity and knowledge of cerebral oxygen saturation monitoring among anesthesiologists in China. The results revealed that the clinical use of cerebral oxygen monitoring in China remains limited, primarily due to the shortage of cerebral oximetry, of which only 67.3% of anesthesiologists reported that their hospitals were equipped with the devices. In addition, clinical practice varied in the anesthesia management process when cerebral desaturation and oxygen saturation occurred. Moreover, anesthesiologists, who usually evaluate POCD after surgery, were familiar with the potential association between POCD and decreased cerebral oxygen saturation.

Cerebral oxygen saturation monitoring is a real-time and non-invasive method to maintain cerebral oxygen balance. It measures the relative proportions of arteriovenous blood and capillary blood components in the target area and takes the weighted average of the arteriovenous blood oxygen saturation in the local brain tissue to reflect the changes in the balance of oxygen supply and oxygen consumption in the brain (12, 13). However, cerebral oximetry has not yet been widely used in operating rooms and intensive care units around the world (14, 15). In the present study, only 67.3% of anesthesiologists in China reported that their hospitals were equipped with cerebral oximetry, despite the fact that our survey might not well reflect the universal use in China. Nevertheless, over 90% of the anesthesiologists had used or were willing to use rSO_2_ monitoring regardless of whether their hospitals were equipped with cerebral oximetry instruments or not. The most likely reason for the low rate of utilization of rSO_2_ monitoring is the accessibility of instruments. Moreover, given the different utilization rates in different surgeries, a lack of guidelines or consensus on the appropriate use of cerebral oximetry may be the reason for its limited use in specialty surgeries during anesthesia. This unconventionally recommended field of use of cerebral oximetry was only common in specific departments, such as neonatology, cardiac, and neurology, which perhaps reflects different levels of confidence concerning the true benefit that cerebral oximetry imparts or institutional practices (16, 17). A global survey of NIRS application in neonatal intensive care units (NICUs) found that only 85 of 235 NICUs owned a NIRS device, but of these, only 9% and 3% used it routinely to guide treatment and prognosis, respectively (18). Apart from a lack of evidence for its clinical guidelines, the relatively high cost of cerebral oximetry, both toward upfront and ongoing costs associated with NIRS technology as well as the monitoring sensors (~$200 per patient), is also of great concern to hospitals and researchers.

The limited utilization of cerebral oxygen monitoring in China may be attributed to various factors. Financial constraints and inadequate insurance coverage may present a burden on patients and anesthesiologists, impeding the widespread adoption of this technique. Insufficient educational and training opportunities might contribute to a lack of awareness and proficiency among practitioners. Additionally, restricted accessibility to cerebral oximetry instruments, especially in secondary hospitals and resource-constrained areas, could hinder implementation. To promote greater utilization, it is necessary to address these barriers by improving insurance policies, enhancing education and training programs, and increasing the availability of equipment. Moreover, comparing international practices and fostering collaborative research efforts could offer valuable insights for overcoming challenges and facilitating the broader adoption of cerebral oxygen monitoring in China.

Although no consensus recommends routine use of cerebral oximetry during anesthesia, evidence from several studies has shown its role in clinical utility, which cannot be ignored (17). Cerebral oximetry-guided cardiac anesthesia, especially during cardiopulmonary bypass, has been shown to significantly reduce mortality and morbidity and is associated with shorter intensive care unit hospital stays (19, 20). In a randomized controlled study of 122 otherwise healthy elderly patients undergoing nonvascular abdominal surgery, control patients (rSO_2_ was monitored but not displayed) who developed intraoperative cerebral desaturation had a lower mini-mental state examination score at the 7th postoperative day and a longer hospital length of stay than patients in the intervention group (rSO_2_ was maintained at ≥75% of the baseline value) (8). For local anesthesia, stump pressure measurement, NIRS, and transcranial Doppler provided similar accuracy for the detection of cerebral ischemia during carotid surgery (21). Our findings suggested that a higher proportion of anesthesiologists who are equipped with cerebral oximetry would consider monitoring rSO_2_ during cardiac surgery (77.4%, *n* = 212) or neurosurgery (40.5%, *n* = 111), whereas none of them use this technology routinely during anesthesia. Similar to our results, a study by Turra et al. investigated the clinical utility of NIRS in cardiac surgery and found that 69.9% of anesthesiologists considered NIRS to be an essential or useful monitor and made treatment decisions based on NIRS (22). These results suggested that anesthesiologists who are willing to use cerebral oximetry expect to achieve better clinical efficacy by real-time monitoring of hypoxia in patients, similar to pulse oximetry. However, there is still some vagueness in how cerebral oximetry guides anesthesia.

A decrease in cerebral oxygen saturation indicates an imbalance in brain oxygen supply, which may result in hypoxic damage to peripheral organs prior to affecting the brain due to disparities in blood supply and oxygen distribution (23, 24). The anesthesiologist needs to perform necessary drugs or physiological interventions to improve hypoxia during anesthesia for preventing or minimizing ischemia damage. Our study found that 90.3% of anesthesiologists would like to adjust anesthetic drugs (isoflurane and propofol) for elevating arterial pressure to ensure adequate blood supply in the brain. However, it is difficult to determine the absolute cutoff value of rSO_2_ for physiological interventions based on the currently available data (19). There is wide intra- and interindividual baseline variability in rSO_2_. The “normal” range lies between 60% and 75%, with a coefficient of variation for absolute baseline values of approximately 10% (25, 26). It has been reported that rSO_2_ <50% or a 20% reduction from the individual baseline is generally considered indicative of the need for intervention (19, 27). In the present survey, most anesthesiologists, based on years of clinical experience, agreed that a 30% reduction from the rSO_2_ baseline requires intervention. Due to the inter-device variability and variability in oxygen saturation targets, this recognized bias might limit the application of the true clinical value of this device, which should be eliminated by compelling evidence from large randomized controlled trials in the future. Therefore, it might be a good practice to use cerebral oximetry as a trend monitor at present.

Geriatric anesthesia is challenging for anesthesiologists in many ways. Many geriatric patients experience age-dependent physiological changes (with or without chronic illness) that increase the risk of POCD, length of hospital stay, treatment costs, and perioperative death (28, 29). All these findings highlight the importance of perioperative anesthesia management in geriatric patients. POCD occurs in 20% to 40% of patients > 60 years of age after major surgery and hospitalization (30). Preventing intraoperative hypotension, especially the maintenance of cerebral perfusion, is indeed important for minimizing the risk of POCD. In several studies, intraoperative declines in rSO_2_ values were associated with the risk of POCD and cognitive changes (31, 32). Our survey found that 96.7% of anesthesiologists agreed that cerebral oxygen monitoring would be helpful for preventing POCD, despite the fact that the data might be greater than actual in practice due to “faking good” or social desirability bias. Importantly, most anesthesiologists hope to have more effective monitoring instruments to help maintain normal for all physiological indicators during anesthesia, reduce the risk of POCD, and improve patient prognosis. This urgent expectation of anesthesiologists for assistance in anesthesia management and postoperative care also prompts the need for large randomized controlled trials to identify the clinical value of NIRS. Recently, a total of six pilot trials have explored the feasibility of cerebral oxygen saturation monitoring for POCD reduction (NCT01839227; NCT03107260; NCT02532530; NCT01149148; NCT02342236; and NCT04714346), and several studies measured the cerebral saturation for assessment of safety of different anesthesia strategies (NCT02967029; NCT01147146; NCT03349658; NCT02327494; NCT03817112; NCT02834845; and NCT03161275).

This study presents some limitations. First, it is difficult to survey all anesthesiologists due to the vast geographical size of China. Selection bias was inevitable because the questionnaires were distributed by the vice chairperson of the Youth Group of the National Society of Anesthesiology across China. Second, there were few responses from anesthesiologists working at secondary hospitals. In addition, multiple anesthesiologists from the same hospital may have filled out the questionnaire, and we cannot comment on the generalizability of these results to those hospitals, which might overrate the number of cerebral oximetry. Third, the questionnaire lacks certain aspects of data, such as the different clinical values of rSO_2_ monitoring in guiding local or general anesthesia and subgroup analyses of specific NIRS usage for cardiovascular surgery, neurosurgery, or orthopedic surgery. Moreover, the rSO_2_ values can be affected by various factors, such as skin pigmentation, extracranial contamination, and physiological conditions (2, 19, 33). Changes in physiological conditions may, in turn, lead to changes in cerebral blood flow or oxygen content. This issue might also influence the decisions of the participants on the necessity of rSO_2_ monitoring. Furthermore, similar studies with well-designed and large samples must be conducted in other countries.

## Conclusion

The survey on rSO_2_ monitoring in geriatric anesthesia indicates a relatively low prevalence of cerebral oximetry utilization, with some heterogeneity in clinical practice and anesthesia management. This study highlights that almost all participants expressed their willingness to use rSO_2_ monitoring in geriatric anesthesia. The limited availability of equipment and relevant knowledge gaps underscore the need for further clinical research to optimize treatment strategies, establish uniform guidelines or recommendations, and provide adequate training.

## Data availability statement

The original contributions presented in the study are included in the article/supplementary material, further inquiries can be directed to the corresponding authors.

## Ethics statement

The studies involving humans were approved by the Medical Ethics Committee of the Second Xiangya Hospital of Central South University (No. 2019S402). The studies were conducted in accordance with the local legislation and institutional requirements. The participants provided their written informed consent to participate in this study.

## Author contributions

XC, YX, and JZ developed the study concept. JZ, HS, and HW performed data extraction. LD and FX performed data analysis and interpretation. JZ and XC wrote the manuscript with critical revision by YX. All authors reviewed and approved the final manuscript.
